# 1,1′-Bis[bis­(4-*tert*-butyl­phen­yl)meth­yl]ferrocene

**DOI:** 10.1107/S1600536812041360

**Published:** 2012-11-03

**Authors:** Heiko Bauer, Yu Sun, Helmut Sitzmann

**Affiliations:** a Department of Chemistry, Technical University of Kaiserslautern, 67663 Kaiserslautern, Germany

## Abstract

The molecule of the title compound, [Fe(C_26_H_31_)_2_], is located on an inversion center. The two cyclopentadienyl rings exhibit a staggered conformation, which results from the bulky bis(4-*tert*-butylphenyl)methyl substituents situated on opposite sides of the molecule.

## Related literature

For reports of the Gomberg radical, see: Gomberg (1900 ▶, 1901 ▶, 1902 ▶). For solution behavior of the triphenyl­methyl radical, see: Lankamp *et al.* (1968 ▶); McBride (1974 ▶). For paramagnetic cyclopentadienyliron complexes, see: Sitzmann *et al.* (1996 ▶); Sitzmann (2001 ▶); Weismann *et al.* (2011 ▶). For cyclo­penta­dienyl radicals, see: Sitzmann *et al.* (1998 ▶, 2000 ▶).
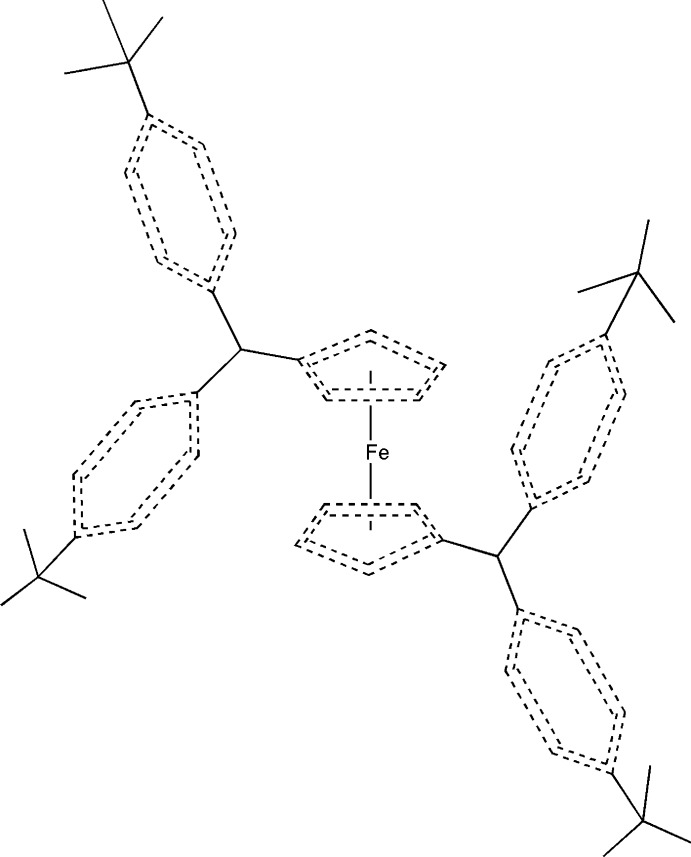


## Experimental

### 

#### Crystal data

[Fe(C_26_H_31_)_2_]
*M* *_r_* = 742.87
Monoclinic, 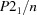

*a* = 6.0893 (2) Å
*b* = 30.7616 (8) Å
*c* = 11.0983 (3) Å
β = 98.982 (3)°
*V* = 2053.40 (10) Å^3^
*Z* = 2
Cu *K*α radiation
μ = 3.19 mm^−1^
*T* = 150 K
0.18 × 0.13 × 0.09 mm

#### Data collection

Oxford Diffraction Xcalibur Sapphire3 Gemini ultra diffractometer
Absorption correction: multi-scan (*CrysAlis PRO*; Oxford Diffraction, 2010) ▶ *T*_min_ = 0.889, *T*_max_ = 1.000
12754 measured reflections
3267 independent reflections
2784 reflections with *I* > 2σ(*I*)
*R*_int_ = 0.028

#### Refinement

*R*[*F* ^2^ > 2σ(*F* ^2^)] = 0.027
*wR*(*F* ^2^) = 0.071
*S* = 1.01
3267 reflections
247 parameters
H-atom parameters constrained
Δρ_max_ = 0.17 e Å^−3^
Δρ_min_ = −0.29 e Å^−3^

### 

Data collection: *CrysAlis CCD* (Oxford Diffraction, 2010 ▶); cell refinement: *CrysAlis CCD*; data reduction: *CrysAlis RED* (Oxford Diffraction, 2010 ▶); program(s) used to solve structure: *SIR92* (Altomare *et al.*, 1994 ▶); program(s) used to refine structure: *SHELXL97* (Sheldrick, 2008 ▶); molecular graphics: *Mercury* (Macrae *et al.*, 2008 ▶); software used to prepare material for publication: *publCIF* (Westrip, 2010 ▶).

## Supplementary Material

Crystal structure: contains datablock(s) I, New_Global_Publ_Block. DOI: 10.1107/S1600536812041360/mw2088sup1.cif

Structure factors: contains datablock(s) I. DOI: 10.1107/S1600536812041360/mw2088Isup2.hkl

Additional supplementary materials:  crystallographic information; 3D view; checkCIF report

## Figures and Tables

**Fig. 1.**
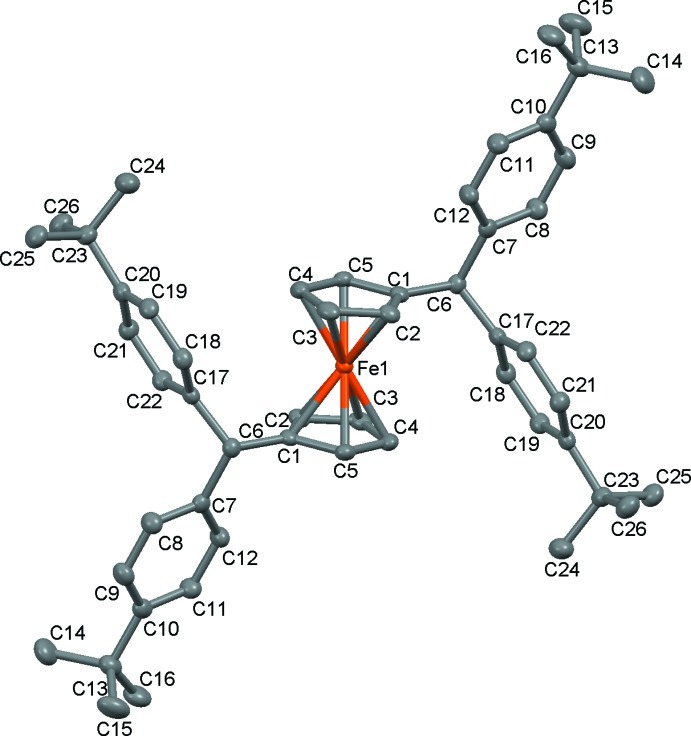
: View of the title compound showing thermal ellipsoids at 50% probability.

**Fig. 2.**
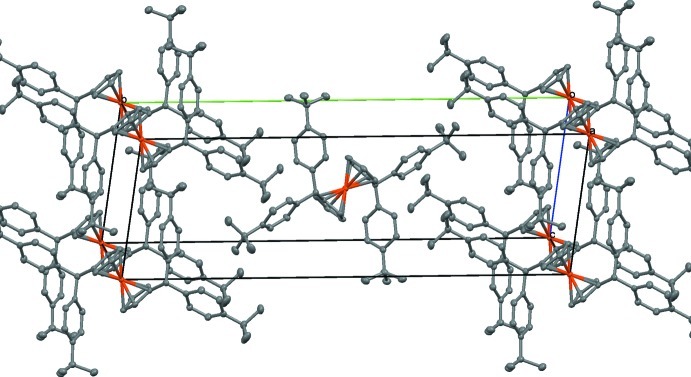
: View of the packing of the title compound.

## supplementary crystallographic information

### Comment

Our interest in paramagnetic cyclopentadienyliron complexes (Sitzmann *et al.*, 1996; Sitzmann, 2001; Weismann *et al.*, 2011) and cyclopentadienyl radicals (Sitzmann *et al.*, 1998; Sitzmann *et al.*, 2000) stimulated experiments aimed at the generation of ferrocenyl derivatives of the Gomberg radical (Gomberg, 1900; Gomberg, 1901; Gomberg, 1902). The triphenylmethyl radical exists in solution in an equilibrium with its unsymmetrical dimer, where one *para* phenyl carbon atom forms a bond to the central methyl carbon of the second molecule (Lankamp *et al.*, 1968; McBride, 1974). In order to prepare starting compounds for the synthesis of ferrocenyl diaryl radicals, diphenylfulvene was equipped with *tert*-butyl substituents in the *para* position to prevent such side reactions. Treatment with lithium aluminium hydride and metalation with *n*-butyllithium, followed by complexation with iron(II) chloride, gave the corresponding ferrocene with two bis(4-*tert*-butylphenyl)methyl substituents.

The iron center is bound to the cyclopentadienyl ring in the typical η^5^ manner. The distance between the two cyclopentadienyl ring planes is 3.315 Å, implying a Cp_cent_—Fe distance of 1.658 Å. The two Cp rings are arranged in a staggered conformation which results from the large di(4-*tert*-butylphenyl)methyl substituents arranging on opposite sides of the molecule.

### Experimental

Synthesis of 5-(di(4-*tert*-butylphenyl)methyl)-1,3-cyclopentadien: To a stirred solution of 1,1'-bis-(4-*tert*-butylphenyl)-(2,4-cyclopentadien-ylidenemethylene) (2.04 g, 6.0 mmol) in THF (60 mL) was added lithium aluminium hydride (300 mg, 8.0 mmol). The reaction mixture was heated at 65 °C for 15 h until the orange suspension became colourless. The colourless suspension was slowly poured onto a mixture of diluted sulfuric acid (10%) and ice. After separation, the aqueous layer was extracted with toluene (3 *x* 100 mL) and the combined organic layers were washed with water (100 mL), dried over MgSO_4_ and taken to dryness *in vacuo*. This yielded a light yellow solid (1.88 g, 5.46 mmol, 91%).

Synthesis of (di(4-*tert*-butylphenyl))methyl-cyclopentadienyl lithium: To a stirred solution of 5-(di(4-*tert*-butylphenyl)methyl)-1,3-cyclopentadien (2.07 g, 6.0 mmol) in diethyl ether (100 mL) at 0 °C a solution (1.9 mol/l) of *n*-butyl lithium (4.0 mL, 6.4 mmol) in hexane was added dropwise. After stirring for ten minutes at 0 °C, the mixture was allowed to stir for 30 minutes at room temperature until the yellow solution turned to an orange suspension. The solvent was removed *in vacuo* and the residue was suspended in pentane (50 mL), filtered and washed with pentane (3 *x* 50 mL), yielding a colourless powder (1.77 g, 5.05 mmol, 84%).

Synthesis of 1,1'-bis[(di(4-*tert*-butylphenyl)methyl)cyclopentadienyl]iron: A mixture of (di(4-*tert*-butylphenyl))methyl-cyclopentadienyl lithium (1.77 g, 5.05 mmol) and anhydrous iron(II) chloride (659.1 mg, 5.20 mmol) in THF (250 mL) was stirred for 18 h at room temperature. The darkened solution was evaporated and the resulting residue extracted with pentane (5 *x* 100 mL). The yellow solution was taken to dryness and the yellow powder recrystallized from pentane. The product could be crystallized at low temperature yielding plate-like yellow crystals (1.16 g, 1.56 mmol, 62%).

### Crystal data

**Table:**

[Fe(C_26_H_31_)_2_]	*F*(000) = 800
*M**_r_* = 742.87	*D*_x_ = 1.201 Mg m^−^^3^
Monoclinic, *P*2_1_/*n*	Cu *K*α radiation, λ = 1.54184 Å
Hall symbol: -P 2yn	Cell parameters from 6229 reflections
*a* = 6.0893 (2) Å	θ = 2.9–62.6°
*b* = 30.7616 (8) Å	µ = 3.19 mm^−^^1^
*c* = 11.0983 (3) Å	*T* = 150 K
β = 98.982 (3)°	Plate, yellow
*V* = 2053.40 (10) Å^3^	0.18 × 0.13 × 0.09 mm
*Z* = 2	

### Data collection

**Table:**

Oxford Diffraction Xcalibur Sapphire3 Gemini ultra diffractometer	3267 independent reflections
Radiation source: Enhance Ultra (Cu) X-ray Source	2784 reflections with *I* > 2σ(*I*)
Mirror monochromator	*R*_int_ = 0.028
Detector resolution: 16.1399 pixels mm^-1^	θ_max_ = 62.7°, θ_min_ = 2.9°
ω scans	*h* = −6→5
Absorption correction: multi-scan (*CrysAlis PRO*; Oxford Diffraction, 2010)	*k* = −31→35
*T*_min_ = 0.889, *T*_max_ = 1.000	*l* = −12→12
12754 measured reflections	

### Refinement

**Table:**

Refinement on *F*^2^	Primary atom site location: structure-invariant direct methods
Least-squares matrix: full	Secondary atom site location: difference Fourier map
*R*[*F*^2^ > 2σ(*F*^2^)] = 0.027	Hydrogen site location: inferred from neighbouring sites
*wR*(*F*^2^) = 0.071	H-atom parameters constrained
*S* = 1.01	*w* = 1/[σ^2^(*F*_o_^2^) + (0.0434*P*)^2^] where *P* = (*F*_o_^2^ + 2*F*_c_^2^)/3
3267 reflections	(Δ/σ)_max_ < 0.001
247 parameters	Δρ_max_ = 0.17 e Å^−^^3^
0 restraints	Δρ_min_ = −0.29 e Å^−^^3^

### Special details

**Table:**

Geometry. All s.u.'s (except the s.u. in the dihedral angle between two l.s. planes) are estimated using the full covariance matrix. The cell s.u.'s are taken into account individually in the estimation of s.u.'s in distances, angles and torsion angles; correlations between s.u.'s in cell parameters are only used when they are defined by crystal symmetry. An approximate (isotropic) treatment of cell s.u.'s is used for estimating s.u.'s involving l.s. planes.
Refinement. Refinement of *F*^2^ against ALL reflections. The weighted *R*-factor *wR* and goodness of fit *S* are based on *F*^2^, conventional *R*-factors *R* are based on *F*, with *F* set to zero for negative *F*^2^. The threshold expression of *F*^2^ > 2σ(*F*^2^) is used only for calculating *R*-factors(gt) *etc*. and is not relevant to the choice of reflections for refinement. *R*-factors based on *F*^2^ are statistically about twice as large as those based on *F*, and *R*- factors based on ALL data will be even larger. All hydrogen atoms were placed in calculated positions (C–H 0.96, 0.98 or 1.00 Å) and refined by using a riding model.

### Fractional atomic coordinates and isotropic or equivalent isotropic displacement parameters (Å^2^)

**Table:**

	*x*	*y*	*z*	*U*_iso_*/*U*_eq_	
C1	0.3111 (2)	0.44424 (5)	0.46953 (13)	0.0208 (3)	
C2	0.5266 (3)	0.43796 (5)	0.43750 (13)	0.0236 (3)	
H2	0.6324	0.4169	0.4717	0.028*	
C3	0.5560 (3)	0.46865 (5)	0.34559 (14)	0.0269 (4)	
H3	0.6848	0.4716	0.3078	0.032*	
C4	0.3609 (3)	0.49414 (5)	0.31997 (13)	0.0265 (4)	
H4	0.3355	0.5171	0.2624	0.032*	
C5	0.2100 (3)	0.47904 (5)	0.39611 (13)	0.0228 (3)	
H5	0.0653	0.4903	0.3978	0.027*	
C6	0.1928 (2)	0.41733 (5)	0.55424 (13)	0.0207 (3)	
H6	0.0580	0.4342	0.5671	0.025*	
C7	0.1099 (2)	0.37397 (5)	0.49700 (13)	0.0213 (3)	
C8	−0.0287 (3)	0.34847 (5)	0.55661 (14)	0.0266 (4)	
H8	−0.0632	0.3579	0.6330	0.032*	
C9	−0.1173 (3)	0.30991 (5)	0.50777 (15)	0.0289 (4)	
H9	−0.2132	0.2937	0.5506	0.035*	
C10	−0.0690 (2)	0.29413 (5)	0.39658 (14)	0.0235 (3)	
C11	0.0726 (3)	0.31913 (5)	0.33893 (14)	0.0274 (4)	
H11	0.1119	0.3092	0.2641	0.033*	
C12	0.1593 (3)	0.35845 (5)	0.38752 (14)	0.0268 (4)	
H12	0.2542	0.3749	0.3446	0.032*	
C13	−0.1713 (3)	0.25134 (5)	0.34417 (15)	0.0291 (4)	
C14	−0.1032 (3)	0.21442 (6)	0.43535 (18)	0.0439 (5)	
H14A	0.0593	0.2127	0.4530	0.066*	
H14B	−0.1631	0.1868	0.4002	0.066*	
H14C	−0.1624	0.2202	0.5110	0.066*	
C15	−0.4254 (3)	0.25543 (6)	0.32226 (19)	0.0446 (5)	
H15A	−0.4774	0.2618	0.3996	0.067*	
H15B	−0.4914	0.2281	0.2888	0.067*	
H15C	−0.4699	0.2791	0.2644	0.067*	
C16	−0.0956 (3)	0.23944 (6)	0.22250 (16)	0.0382 (4)	
H16A	−0.1366	0.2628	0.1633	0.057*	
H16B	−0.1679	0.2124	0.1912	0.057*	
H16C	0.0661	0.2355	0.2354	0.057*	
C17	0.3308 (2)	0.41091 (5)	0.67958 (13)	0.0211 (3)	
C18	0.2846 (3)	0.43382 (5)	0.78101 (14)	0.0240 (3)	
H18	0.1578	0.4522	0.7734	0.029*	
C19	0.4209 (3)	0.43015 (5)	0.89289 (13)	0.0245 (4)	
H19	0.3853	0.4462	0.9604	0.029*	
C20	0.6087 (3)	0.40365 (5)	0.90922 (13)	0.0226 (3)	
C21	0.6488 (3)	0.37976 (5)	0.80823 (13)	0.0244 (3)	
H21	0.7732	0.3608	0.8162	0.029*	
C22	0.5115 (3)	0.38301 (5)	0.69618 (14)	0.0240 (3)	
H22	0.5421	0.3658	0.6297	0.029*	
C23	0.7610 (3)	0.40157 (5)	1.03284 (14)	0.0260 (4)	
C24	0.8546 (3)	0.44714 (6)	1.06587 (15)	0.0358 (4)	
H24A	0.9356	0.4575	1.0017	0.054*	
H24B	0.9560	0.4459	1.1436	0.054*	
H24C	0.7321	0.4671	1.0733	0.054*	
C25	0.6265 (3)	0.38601 (6)	1.13129 (14)	0.0320 (4)	
H25A	0.5061	0.4066	1.1373	0.048*	
H25B	0.7245	0.3842	1.2101	0.048*	
H25C	0.5635	0.3573	1.1091	0.048*	
C26	0.9574 (3)	0.37061 (6)	1.03084 (15)	0.0335 (4)	
H26A	0.9017	0.3410	1.0130	0.050*	
H26B	1.0530	0.3711	1.1105	0.050*	
H26C	1.0432	0.3799	0.9677	0.050*	
Fe1	0.5000	0.5000	0.5000	0.01935 (11)	

### Atomic displacement parameters (Å^2^)

**Table:**

	*U*^11^	*U*^22^	*U*^33^	*U*^12^	*U*^13^	*U*^23^
C1	0.0256 (8)	0.0166 (8)	0.0194 (8)	−0.0032 (6)	0.0008 (6)	−0.0040 (6)
C2	0.0272 (9)	0.0183 (8)	0.0250 (8)	−0.0019 (7)	0.0036 (6)	−0.0059 (6)
C3	0.0333 (9)	0.0273 (9)	0.0213 (8)	−0.0084 (7)	0.0079 (7)	−0.0092 (7)
C4	0.0352 (9)	0.0256 (9)	0.0169 (8)	−0.0089 (7)	−0.0016 (6)	0.0000 (6)
C5	0.0235 (8)	0.0213 (8)	0.0215 (8)	−0.0021 (6)	−0.0028 (6)	−0.0027 (6)
C6	0.0225 (8)	0.0182 (8)	0.0214 (8)	0.0021 (6)	0.0031 (6)	0.0005 (6)
C7	0.0212 (8)	0.0201 (8)	0.0215 (8)	0.0025 (6)	−0.0002 (6)	0.0018 (6)
C8	0.0308 (9)	0.0257 (9)	0.0245 (9)	−0.0020 (7)	0.0082 (7)	−0.0023 (7)
C9	0.0291 (9)	0.0254 (9)	0.0332 (9)	−0.0050 (7)	0.0079 (7)	0.0009 (7)
C10	0.0227 (8)	0.0192 (8)	0.0270 (8)	0.0026 (7)	−0.0014 (6)	0.0005 (6)
C11	0.0334 (9)	0.0253 (9)	0.0235 (8)	−0.0021 (7)	0.0044 (7)	−0.0035 (7)
C12	0.0334 (9)	0.0239 (9)	0.0235 (8)	−0.0062 (7)	0.0056 (7)	0.0000 (7)
C13	0.0302 (9)	0.0210 (8)	0.0347 (9)	−0.0017 (7)	0.0007 (7)	−0.0029 (7)
C14	0.0592 (13)	0.0221 (9)	0.0485 (12)	−0.0034 (9)	0.0023 (10)	0.0008 (8)
C15	0.0308 (11)	0.0397 (11)	0.0609 (13)	−0.0070 (9)	−0.0006 (9)	−0.0137 (9)
C16	0.0429 (11)	0.0280 (10)	0.0424 (11)	−0.0061 (8)	0.0023 (8)	−0.0117 (8)
C17	0.0246 (8)	0.0182 (8)	0.0209 (8)	−0.0047 (6)	0.0044 (6)	0.0000 (6)
C18	0.0271 (9)	0.0206 (8)	0.0253 (8)	0.0008 (7)	0.0073 (7)	−0.0002 (6)
C19	0.0327 (9)	0.0218 (8)	0.0198 (8)	−0.0009 (7)	0.0066 (7)	−0.0024 (6)
C20	0.0281 (9)	0.0196 (8)	0.0207 (8)	−0.0035 (7)	0.0058 (6)	0.0015 (6)
C21	0.0269 (9)	0.0221 (8)	0.0237 (8)	0.0029 (7)	0.0028 (6)	0.0005 (6)
C22	0.0315 (9)	0.0197 (8)	0.0210 (8)	0.0011 (7)	0.0048 (6)	−0.0024 (6)
C23	0.0298 (9)	0.0276 (9)	0.0199 (8)	−0.0025 (7)	0.0018 (6)	−0.0003 (7)
C24	0.0411 (11)	0.0337 (10)	0.0308 (9)	−0.0081 (8)	−0.0006 (8)	−0.0033 (8)
C25	0.0383 (10)	0.0363 (10)	0.0212 (8)	−0.0017 (8)	0.0038 (7)	0.0010 (7)
C26	0.0343 (10)	0.0394 (10)	0.0254 (9)	0.0020 (8)	−0.0001 (7)	0.0023 (8)
Fe1	0.02376 (19)	0.01684 (18)	0.01674 (17)	−0.00199 (15)	0.00099 (12)	−0.00149 (14)

### Geometric parameters (Å, º)

**Table:**

C1—C2	1.425 (2)	C15—H15A	0.9800
C1—C5	1.425 (2)	C15—H15B	0.9800
C1—C6	1.516 (2)	C15—H15C	0.9800
C1—Fe1	2.0634 (14)	C16—H16A	0.9800
C2—C3	1.421 (2)	C16—H16B	0.9800
C2—Fe1	2.0455 (15)	C16—H16C	0.9800
C2—H2	0.9500	C17—C22	1.385 (2)
C3—C4	1.415 (2)	C17—C18	1.394 (2)
C3—Fe1	2.0409 (15)	C18—C19	1.386 (2)
C3—H3	0.9500	C18—H18	0.9500
C4—C5	1.420 (2)	C19—C20	1.393 (2)
C4—Fe1	2.0527 (15)	C19—H19	0.9500
C4—H4	0.9500	C20—C21	1.393 (2)
C5—Fe1	2.0560 (14)	C20—C23	1.533 (2)
C5—H5	0.9500	C21—C22	1.390 (2)
C6—C17	1.522 (2)	C21—H21	0.9500
C6—C7	1.529 (2)	C22—H22	0.9500
C6—H6	1.0000	C23—C26	1.532 (2)
C7—C12	1.382 (2)	C23—C24	1.536 (2)
C7—C8	1.393 (2)	C23—C25	1.541 (2)
C8—C9	1.379 (2)	C24—H24A	0.9800
C8—H8	0.9500	C24—H24B	0.9800
C9—C10	1.399 (2)	C24—H24C	0.9800
C9—H9	0.9500	C25—H25A	0.9800
C10—C11	1.385 (2)	C25—H25B	0.9800
C10—C13	1.531 (2)	C25—H25C	0.9800
C11—C12	1.394 (2)	C26—H26A	0.9800
C11—H11	0.9500	C26—H26B	0.9800
C12—H12	0.9500	C26—H26C	0.9800
C13—C15	1.534 (2)	Fe1—C3^i^	2.0409 (15)
C13—C14	1.535 (2)	Fe1—C2^i^	2.0455 (15)
C13—C16	1.538 (2)	Fe1—C4^i^	2.0527 (15)
C14—H14A	0.9800	Fe1—C5^i^	2.0560 (15)
C14—H14B	0.9800	Fe1—C1^i^	2.0634 (15)
C14—H14C	0.9800		
			
C2—C1—C5	106.94 (13)	C17—C18—H18	119.5
C2—C1—C6	128.72 (13)	C18—C19—C20	121.92 (14)
C5—C1—C6	124.05 (14)	C18—C19—H19	119.0
C2—C1—Fe1	69.04 (8)	C20—C19—H19	119.0
C5—C1—Fe1	69.48 (8)	C19—C20—C21	116.58 (14)
C6—C1—Fe1	131.21 (10)	C19—C20—C23	120.70 (13)
C3—C2—C1	108.24 (14)	C21—C20—C23	122.72 (14)
C3—C2—Fe1	69.47 (9)	C22—C21—C20	121.70 (15)
C1—C2—Fe1	70.38 (8)	C22—C21—H21	119.2
C3—C2—H2	125.9	C20—C21—H21	119.2
C1—C2—H2	125.9	C17—C22—C21	121.21 (14)
Fe1—C2—H2	125.8	C17—C22—H22	119.4
C4—C3—C2	108.45 (14)	C21—C22—H22	119.4
C4—C3—Fe1	70.23 (8)	C26—C23—C20	112.19 (13)
C2—C3—Fe1	69.82 (8)	C26—C23—C24	108.03 (14)
C4—C3—H3	125.8	C20—C23—C24	109.01 (13)
C2—C3—H3	125.8	C26—C23—C25	108.64 (13)
Fe1—C3—H3	125.8	C20—C23—C25	109.48 (13)
C3—C4—C5	107.45 (14)	C24—C23—C25	109.45 (13)
C3—C4—Fe1	69.33 (8)	C23—C24—H24A	109.5
C5—C4—Fe1	69.91 (8)	C23—C24—H24B	109.5
C3—C4—H4	126.3	H24A—C24—H24B	109.5
C5—C4—H4	126.3	C23—C24—H24C	109.5
Fe1—C4—H4	126.1	H24A—C24—H24C	109.5
C4—C5—C1	108.92 (14)	H24B—C24—H24C	109.5
C4—C5—Fe1	69.66 (8)	C23—C25—H25A	109.5
C1—C5—Fe1	70.03 (8)	C23—C25—H25B	109.5
C4—C5—H5	125.5	H25A—C25—H25B	109.5
C1—C5—H5	125.5	C23—C25—H25C	109.5
Fe1—C5—H5	126.3	H25A—C25—H25C	109.5
C1—C6—C17	112.94 (12)	H25B—C25—H25C	109.5
C1—C6—C7	112.14 (12)	C23—C26—H26A	109.5
C17—C6—C7	111.48 (12)	C23—C26—H26B	109.5
C1—C6—H6	106.6	H26A—C26—H26B	109.5
C17—C6—H6	106.6	C23—C26—H26C	109.5
C7—C6—H6	106.6	H26A—C26—H26C	109.5
C12—C7—C8	117.03 (14)	H26B—C26—H26C	109.5
C12—C7—C6	124.30 (14)	C3^i^—Fe1—C3	180.00 (8)
C8—C7—C6	118.65 (13)	C3^i^—Fe1—C2^i^	40.71 (6)
C9—C8—C7	121.78 (15)	C3—Fe1—C2^i^	139.29 (6)
C9—C8—H8	119.1	C3^i^—Fe1—C2	139.29 (6)
C7—C8—H8	119.1	C3—Fe1—C2	40.71 (6)
C8—C9—C10	121.49 (15)	C2^i^—Fe1—C2	180.00 (3)
C8—C9—H9	119.3	C3^i^—Fe1—C4^i^	40.43 (6)
C10—C9—H9	119.3	C3—Fe1—C4^i^	139.57 (6)
C11—C10—C9	116.52 (14)	C2^i^—Fe1—C4^i^	68.31 (6)
C11—C10—C13	123.20 (14)	C2—Fe1—C4^i^	111.69 (6)
C9—C10—C13	120.29 (14)	C3^i^—Fe1—C4	139.57 (6)
C10—C11—C12	121.92 (15)	C3—Fe1—C4	40.43 (6)
C10—C11—H11	119.0	C2^i^—Fe1—C4	111.69 (6)
C12—C11—H11	119.0	C2—Fe1—C4	68.31 (6)
C7—C12—C11	121.24 (15)	C4^i^—Fe1—C4	180.000 (1)
C7—C12—H12	119.4	C3^i^—Fe1—C5^i^	67.80 (6)
C11—C12—H12	119.4	C3—Fe1—C5^i^	112.20 (6)
C10—C13—C15	109.28 (14)	C2^i^—Fe1—C5^i^	67.90 (6)
C10—C13—C14	109.49 (13)	C2—Fe1—C5^i^	112.10 (6)
C15—C13—C14	109.10 (15)	C4^i^—Fe1—C5^i^	40.43 (6)
C10—C13—C16	112.22 (14)	C4—Fe1—C5^i^	139.57 (6)
C15—C13—C16	108.29 (14)	C3^i^—Fe1—C5	112.20 (6)
C14—C13—C16	108.40 (14)	C3—Fe1—C5	67.80 (6)
C13—C14—H14A	109.5	C2^i^—Fe1—C5	112.10 (6)
C13—C14—H14B	109.5	C2—Fe1—C5	67.90 (6)
H14A—C14—H14B	109.5	C4^i^—Fe1—C5	139.57 (6)
C13—C14—H14C	109.5	C4—Fe1—C5	40.43 (6)
H14A—C14—H14C	109.5	C5^i^—Fe1—C5	180.00 (8)
H14B—C14—H14C	109.5	C3^i^—Fe1—C1^i^	68.37 (6)
C13—C15—H15A	109.5	C3—Fe1—C1^i^	111.63 (6)
C13—C15—H15B	109.5	C2^i^—Fe1—C1^i^	40.58 (6)
H15A—C15—H15B	109.5	C2—Fe1—C1^i^	139.42 (6)
C13—C15—H15C	109.5	C4^i^—Fe1—C1^i^	68.45 (6)
H15A—C15—H15C	109.5	C4—Fe1—C1^i^	111.55 (6)
H15B—C15—H15C	109.5	C5^i^—Fe1—C1^i^	40.49 (6)
C13—C16—H16A	109.5	C5—Fe1—C1^i^	139.51 (6)
C13—C16—H16B	109.5	C3^i^—Fe1—C1	111.63 (6)
H16A—C16—H16B	109.5	C3—Fe1—C1	68.37 (6)
C13—C16—H16C	109.5	C2^i^—Fe1—C1	139.42 (6)
H16A—C16—H16C	109.5	C2—Fe1—C1	40.58 (6)
H16B—C16—H16C	109.5	C4^i^—Fe1—C1	111.55 (6)
C22—C17—C18	117.55 (14)	C4—Fe1—C1	68.45 (6)
C22—C17—C6	121.06 (13)	C5^i^—Fe1—C1	139.51 (6)
C18—C17—C6	121.36 (14)	C5—Fe1—C1	40.49 (6)
C19—C18—C17	120.93 (15)	C1^i^—Fe1—C1	180.0
C19—C18—H18	119.5		
			
C5—C1—C2—C3	0.02 (16)	C2—C3—Fe1—C5^i^	98.56 (10)
C6—C1—C2—C3	173.97 (14)	C4—C3—Fe1—C5	37.93 (9)
Fe1—C1—C2—C3	−59.37 (10)	C2—C3—Fe1—C5	−81.44 (10)
C5—C1—C2—Fe1	59.39 (10)	C4—C3—Fe1—C1^i^	−98.28 (9)
C6—C1—C2—Fe1	−126.66 (15)	C2—C3—Fe1—C1^i^	142.36 (9)
C1—C2—C3—C4	0.11 (16)	C4—C3—Fe1—C1	81.72 (9)
Fe1—C2—C3—C4	−59.84 (10)	C2—C3—Fe1—C1	−37.64 (9)
C1—C2—C3—Fe1	59.94 (10)	C3—C2—Fe1—C3^i^	180.000 (1)
C2—C3—C4—C5	−0.19 (17)	C1—C2—Fe1—C3^i^	60.78 (13)
Fe1—C3—C4—C5	−59.77 (10)	C1—C2—Fe1—C3	−119.22 (13)
C2—C3—C4—Fe1	59.58 (10)	C3—C2—Fe1—C4^i^	−142.53 (9)
C3—C4—C5—C1	0.20 (17)	C1—C2—Fe1—C4^i^	98.24 (9)
Fe1—C4—C5—C1	−59.20 (10)	C3—C2—Fe1—C4	37.47 (9)
C3—C4—C5—Fe1	59.40 (10)	C1—C2—Fe1—C4	−81.76 (9)
C2—C1—C5—C4	−0.14 (16)	C3—C2—Fe1—C5^i^	−98.81 (10)
C6—C1—C5—C4	−174.44 (13)	C1—C2—Fe1—C5^i^	141.97 (9)
Fe1—C1—C5—C4	58.98 (10)	C3—C2—Fe1—C5	81.19 (10)
C2—C1—C5—Fe1	−59.11 (10)	C1—C2—Fe1—C5	−38.03 (9)
C6—C1—C5—Fe1	126.59 (14)	C3—C2—Fe1—C1^i^	−60.78 (13)
C2—C1—C6—C17	53.1 (2)	C1—C2—Fe1—C1^i^	180.0
C5—C1—C6—C17	−133.93 (14)	C3—C2—Fe1—C1	119.22 (13)
Fe1—C1—C6—C17	−42.25 (19)	C3—C4—Fe1—C3^i^	180.000 (1)
C2—C1—C6—C7	−73.92 (18)	C5—C4—Fe1—C3^i^	−61.36 (13)
C5—C1—C6—C7	99.09 (17)	C5—C4—Fe1—C3	118.64 (13)
Fe1—C1—C6—C7	−169.23 (11)	C3—C4—Fe1—C2^i^	142.29 (9)
C1—C6—C7—C12	7.5 (2)	C5—C4—Fe1—C2^i^	−99.07 (10)
C17—C6—C7—C12	−120.27 (16)	C3—C4—Fe1—C2	−37.71 (9)
C1—C6—C7—C8	−171.08 (13)	C5—C4—Fe1—C2	80.93 (10)
C17—C6—C7—C8	61.17 (18)	C3—C4—Fe1—C5^i^	61.36 (13)
C12—C7—C8—C9	−1.4 (2)	C5—C4—Fe1—C5^i^	180.0
C6—C7—C8—C9	177.24 (14)	C3—C4—Fe1—C5	−118.64 (13)
C7—C8—C9—C10	1.1 (3)	C3—C4—Fe1—C1^i^	98.48 (10)
C8—C9—C10—C11	0.3 (2)	C5—C4—Fe1—C1^i^	−142.88 (9)
C8—C9—C10—C13	−179.54 (15)	C3—C4—Fe1—C1	−81.52 (10)
C9—C10—C11—C12	−1.3 (2)	C5—C4—Fe1—C1	37.12 (9)
C13—C10—C11—C12	178.54 (15)	C4—C5—Fe1—C3^i^	142.07 (9)
C8—C7—C12—C11	0.4 (2)	C1—C5—Fe1—C3^i^	−97.77 (10)
C6—C7—C12—C11	−178.15 (14)	C4—C5—Fe1—C3	−37.93 (9)
C10—C11—C12—C7	1.0 (3)	C1—C5—Fe1—C3	82.23 (10)
C11—C10—C13—C15	−119.29 (17)	C4—C5—Fe1—C2^i^	97.95 (10)
C9—C10—C13—C15	60.6 (2)	C1—C5—Fe1—C2^i^	−141.88 (9)
C11—C10—C13—C14	121.27 (17)	C4—C5—Fe1—C2	−82.05 (10)
C9—C10—C13—C14	−58.9 (2)	C1—C5—Fe1—C2	38.12 (9)
C11—C10—C13—C16	0.8 (2)	C4—C5—Fe1—C4^i^	180.0
C9—C10—C13—C16	−179.29 (14)	C1—C5—Fe1—C4^i^	−59.83 (13)
C1—C6—C17—C22	−73.47 (18)	C1—C5—Fe1—C4	120.17 (13)
C7—C6—C17—C22	53.85 (19)	C4—C5—Fe1—C1^i^	59.83 (13)
C1—C6—C17—C18	104.54 (16)	C1—C5—Fe1—C1^i^	180.0
C7—C6—C17—C18	−128.14 (15)	C4—C5—Fe1—C1	−120.17 (13)
C22—C17—C18—C19	2.8 (2)	C2—C1—Fe1—C3^i^	−142.24 (9)
C6—C17—C18—C19	−175.22 (14)	C5—C1—Fe1—C3^i^	99.29 (10)
C17—C18—C19—C20	−0.1 (2)	C6—C1—Fe1—C3^i^	−18.54 (16)
C18—C19—C20—C21	−2.1 (2)	C2—C1—Fe1—C3	37.76 (9)
C18—C19—C20—C23	177.73 (14)	C5—C1—Fe1—C3	−80.71 (10)
C19—C20—C21—C22	1.5 (2)	C6—C1—Fe1—C3	161.46 (16)
C23—C20—C21—C22	−178.33 (14)	C2—C1—Fe1—C2^i^	180.0
C18—C17—C22—C21	−3.5 (2)	C5—C1—Fe1—C2^i^	61.54 (12)
C6—C17—C22—C21	174.62 (14)	C6—C1—Fe1—C2^i^	−56.30 (17)
C20—C21—C22—C17	1.3 (2)	C5—C1—Fe1—C2	−118.46 (12)
C19—C20—C23—C26	−179.90 (14)	C6—C1—Fe1—C2	123.70 (17)
C21—C20—C23—C26	−0.1 (2)	C2—C1—Fe1—C4^i^	−98.61 (9)
C19—C20—C23—C24	−60.29 (19)	C5—C1—Fe1—C4^i^	142.93 (9)
C21—C20—C23—C24	119.54 (16)	C6—C1—Fe1—C4^i^	25.09 (16)
C19—C20—C23—C25	59.40 (19)	C2—C1—Fe1—C4	81.39 (9)
C21—C20—C23—C25	−120.76 (16)	C5—C1—Fe1—C4	−37.07 (9)
C4—C3—Fe1—C2^i^	−60.63 (13)	C6—C1—Fe1—C4	−154.91 (16)
C2—C3—Fe1—C2^i^	180.000 (1)	C2—C1—Fe1—C5^i^	−61.54 (12)
C4—C3—Fe1—C2	119.37 (13)	C5—C1—Fe1—C5^i^	180.0
C4—C3—Fe1—C4^i^	180.000 (1)	C6—C1—Fe1—C5^i^	62.17 (18)
C2—C3—Fe1—C4^i^	60.63 (13)	C2—C1—Fe1—C5	118.46 (12)
C2—C3—Fe1—C4	−119.37 (13)	C6—C1—Fe1—C5	−117.83 (18)
C4—C3—Fe1—C5^i^	−142.07 (9)		

Symmetry code: (i) −*x*+1, −*y*+1, −*z*+1.
